# Incidence of cognitive impairment and dementia after hospitalisation for pneumonia: a UK population-based matched cohort study

**DOI:** 10.1183/23120541.00328-2022

**Published:** 2023-05-09

**Authors:** Christos V. Chalitsios, Vadsala Baskaran, Rowan H. Harwood, Wei Shen Lim, Tricia M. McKeever

**Affiliations:** 1 Division of Epidemiology and Public Health, School of Medicine, University of Nottingham, Nottingham, UK; 2 Geriatric Medicine, Nottingham University Hospitals NHS Trust, Nottingham, UK; 3 School of Health Sciences, University of Nottingham, Nottingham, UK; 4 Respiratory Medicine, Nottingham University Hospitals NHS Trust, Nottingham, UK

## Abstract

**Background:**

Survivors of common infections may develop cognitive impairment or dementia; however, the risk of these conditions in people hospitalised with pneumonia is not well established.

**Methods:**

A matched cohort study was conducted using Hospital Episode Statistics (HES) data linked to the Clinical Practice Research Database (CPRD). Adults with the first International Classification of Diseases (10th Revision) code for pneumonia recorded in the HES between 1 July 2002 and 30 June 2017 were included, and up to four controls without hospitalisation for pneumonia in the CPRD were matched by sex, age and practice. Cognitive impairment and dementia incidence rates were calculated and survival analysis was performed comparing those hospitalised with pneumonia to the general population.

**Results:**

The incidence rates of cognitive impairment and dementia were 18 (95% CI 17.3–18.7) and 13.2 (95% CI 13–13.5) per 1000 person-years among persons previously hospitalised with pneumonia and the matched cohort respectively. People previously hospitalised with pneumonia had 53% higher incidence of cognitive impairment and dementia (adjusted hazard ratio (aHR) 1.53, 95% CI 1.46–1.61) than their matched cohort. The highest incidence was observed within 1 year of hospitalisation for pneumonia compared to the general population (aHR 1.89, 95% CI 1.75–2.05). Age modified the effect of hospitalisation for pneumonia on cognitive impairment and dementia such that the size of effect was stronger in people between 45 and 60 years old (p-value for interaction <0.0001).

**Conclusion:**

Cognitive impairment and dementia are more likely to be diagnosed in people who have been hospitalised for pneumonia, especially in the first year after discharge, than in the general population.

## Abstract 2

**Cognitive impairment and dementia are more likely to be diagnosed in people who have been hospitalised for pneumonia, especially in the first year after discharge, than in the general population** https://bit.ly/3Dxd2PB

## Introduction

Pneumonia is a major cause of morbidity and mortality worldwide [1]. ∼1% of the UK adult population develops pneumonia annually and it accounts for >100 000 hospital admissions every year [2, 3]. Although pneumonia is an acute illness, substantial longer-term associated risks are recognised: >50% mortality after a 5-year period and an almost two-fold greater risk of subsequent pneumonia compared to those without previous pneumonia [1]. Additionally, despite major advances in risk stratification and management, health sequelae in survivors of pneumonia have not significantly improved over recent years [4].

Common infections are an established risk factor for acute cognitive impairment in older adults (≥65 years) and increasing evidence from longitudinal studies suggests that they are also associated with an increased risk of dementia [5]. Studies also report that survivors of critical illness are at increased risk of developing cognitive impairment even many years after discharge from the hospital and intensive care unit [6–9]. The current literature has mainly focused on outcomes such as mortality and cardiovascular diseases after pneumonia [1, 10, 11]. A small number of studies focusing on older persons have reported that pneumonia survivors experience diminished cognition [12–14]. However, these studies did not include adults across all ages.

The aim of this study was to address the gap in knowledge regarding the risk of incident cognitive impairment and dementia in people hospitalised with pneumonia when compared to the general population.

## Methods

### Source population

A matched cohort study was conducted using hospitalisation data from Hospital Episode Statistics (HES) linked to the Clinical Practice Research Datalink (CPRD), a large longitudinal UK primary care database that provides anonymised electronic health records from general practices. HES admitted patient care data contained details of all admissions to National Health Service hospitals in England from 1 April 1997 to 30 November 2018, with diagnostic data coded using International Classification of Diseases, 10th Revision (ICD-10).

### Study population and outcomes

Adults (≥18 years old) with the first hospitalisation for pneumonia (index date) recorded in the HES between 1 July 2002 and 30 June 2017 were included. Pneumonia (ICD-10 codes J12–J18) was defined as the primary code for the first episode of hospitalisation (table S1). Patients were excluded if they 1) had been registered at the practice for <1 year before admission [15], 2) had hospital-acquired pneumonia (admission for ≥1 day in the 10 days preceding the index admission) or 3) had recorded pre-existing cognitive impairment or dementia. Up to four randomly selected people without hospitalisation with pneumonia in CPRD were matched with each person hospitalised with pneumonia based on sex, age (±1 year) and general practice. Controls were assigned the same index date as their matched people hospitalised for pneumonia. Participants were eligible for inclusion if their record was labelled as acceptable according to CPRD recommendations.

The outcomes of interest were the time from the index date to the first read coded cognitive impairment and dementia, both combined and separately (tables S2 and S3). The set of codes used was based on previously published lists [7, 16, 17] augmented by a manual search of all read terms within the CPRD. An expert geriatrician (R.H. Harwood) was consulted to help identify all relevant read codes of the outcomes.

### Follow-up

All patients were followed up from day 1 after the date of discharge from hospital to either the date of outcome of interest, end of data collection (30 June 2017), date of transfer out of practice or date of last data collection for the practice or date of death, whichever came first.

### Statistical analysis

The power of the study size was calculated to detect the increased risk of cognitive impairment and dementia when compared to the general population. The baseline characteristics between people hospitalised for pneumonia and controls were compared by performing conditional logistic regression using the matched set as the stratum variable. Incidence rates of cognitive impairment and dementia were calculated by dividing the number of incident diagnosis by follow-up person-years for both groups. The probability of experiencing the outcomes during the follow-up time was presented with a plot using the Kaplan–Meier method and the log-rank test examined any difference between the groups. Performing a Cox regression analysis, stratified by matched set, we calculated the hazard ratio (HR) estimates and 95% confidence intervals comparing the cognitive impairment and dementia risk between hospitalised-for-pneumonia and control patients. Directed acyclic graphs (DAG) help in depicting the status of the covariates in a statistical model (confounders, mediators and colliders). DAGs are based on a good understanding of the literature and are made prior to any analyses to determine which variables are appropriate to include in the adjustment of the model [18]. Following a review of published literature [19], a DAG was used to identify the minimum sufficient adjustment set of confounders (figure S1). This included age at the index date, sex including only those clearly classified as male or female, smoking, body mass index (BMI), alcohol consumption and comorbidities (depression, cerebrovascular diseases, type II diabetes, hypertension and traumatic brain injury). Comorbidities were also summarised using the Charlson comorbidity index score [20]. Validated code lists were used for these confounders [21–23]. The Cox model assumption was tested using Schoenfeld residuals. Incidence rates and HRs by follow-up intervals were additionally determined: 0–1 year, >1–4 years and >4–16 years. A new category was assigned to missing data for smoking, BMI and alcohol consumption. In addition, two more analyses were conducted excluding those with missing data and imputing the missing data with multiple imputation. Five imputations were generated, and the imputed model consisted of age, sex, outcome, case and all confounders. A subgroup analysis by sex and age group was also performed. Age and sex were examined by fitting interaction terms (age by case and sex by case) and carrying out a likelihood ratio test to determine whether they modified the effect of pneumonia on cognitive impairment and dementia. All data management and statistical analysis were performed using R version 4.1.1.

## Results

### Baseline characteristics

The study cohort comprised 55 808 persons hospitalised with pneumonia and 206 108 age-, sex- and practice-matched controls without pneumonia (table 1). The median age was 75 years (interquartile range (IQR) 61–84 years) and 74 years (IQR 59–83 years) for people hospitalised for pneumonia and controls, respectively. Median follow-up was 1.7 years (IQR 0.5–4 years) in people hospitalised for pneumonia and 3 years (IQR 1.4–5.7 years) in controls. More persons hospitalised for pneumonia were current smokers than controls (21.1% *versus* 13.4%, p<0.0001) and had more comorbid diseases (74.1% *versus* 51.5%, p<0.0001).

**TABLE 1:** Baseline characteristics of those patients hospitalised for pneumonia and a matched comparison cohort of controls

**Characteristic**	**Hospitalisation for pneumonia**	**Controls**
**Patients**	55 808	206 168
**Follow-up, years**		
Mean±sd	2.7±2.9	3.9±3.2
Median (IQR)	1.7 (0.5–4)	3 (1.4–5.7)
**Age, years**		
Mean±sd	70.6±17.8	69.6±17.8
Median (IQR)	75 (61–84)	74 (59–83)
18–44	6125 (11%)	24 163 (11.7%)
45–60	7633 (13.7%)	30 111 (14.6%)
61–74	13 452 (24.1%)	51 917 (25.2%)
75–83	13 461 (24.1%)	50 029 (24.3%)
>83	15 137 (27.1%)	59 948 (24.2%)
**Sex**		
Male	27 699 (49.6%)	101 638 (49.3%)
Female	28 109 (50.4%)	104 530 (50.7%)
**Body mass index, kg·m^−2^**		
Mean±sd	26.4±6.4	26.8±5.3
Median (IQR)	25.6 (22.1–29.8)	26.2 (23.3–29.7)
Underweight^#^	3375 (6%)	4876 (2.4%)
Normal^¶^	17 377 (31.2%)	58 939 (28.6%)
Overweight^+^	13 721 (24.6%)	60 971 (29.6%)
Obese^§^	11 051 (19.8%)	37 990 (18.4%)
Unknown	10 273 (18.4%)	43 392 (21%)
**Smoking status**		
Never-smokers	21 887 (39.2%)	106 064 (51.5%)
Ex-smokers	20 474 (36.7%)	59 978 (29.1%)
Current smokers	11 753 (21.1%)	27 695 (13.4%)
Unknown	1694 (3%)	12 431 (6%)
**Alcohol status**		
Never	11 868 (21.3%)	35 326 (17.1%)
Occasional	7658 (13.7%)	29 339 (14.2%)
Current	26 881 (48.2%)	105 996 (51.4%)
Ex-drinkers	2551 (4.6%)	5403 (2.6%)
Unknown	6850 (12.3%)	30 104 (14.6%)
**Charlson comorbidity index**		
0	14 469 (25.9%)	100 087 (48.5%)
1	12 007 (21.5%)	37 139 (18%)
2	9708 (17.4%)	30 097 (14.6%)
3	7558 (13.5%)	18 268 (8.9%)
4	4852 (8.7%)	9771 (4.7%)
≥5	7220 (12.9%)	10 809 (5.2%)
**Comorbidities**		
Depression	11 367 (20.4%)	28 404 (13.8%)
Hypertension	20 218 (36.2%)	69 681 (33.8%)
Type II diabetes	9149 (16.4%)	22 777 (11%)
Cerebrovascular disease	5617 (10%)	11 606 (5.4%)
Traumatic brain injury	377 (0.68%)	969 (0.47%)

Percentages have been rounded and might not total 100. IQR: interquartile range. p<0.0001 for the baseline characteristics between the two groups except age and sex, in which there are no significant differences. ^#^: <18.5 kg·m^−2^; ^¶^: 18.5–24.9 kg·m^−2^; ^+^: 25–29.9 kg·m^−2^; ^§^: ≥30 kg·m^−2^.

### Risk of cognitive impairment and dementia

During the whole study period, the crude incidence of cognitive impairment and dementia was higher in people hospitalised for pneumonia than the controls (figure S2) with the incidence rates of 18 (95% CI 17.3–18.7) and 13.2 (95% CI 13–13.5) per 1000 person-years, respectively (table 2). The Kaplan–Meier plot also demonstrated a significantly higher probability of cognitive impairment and dementia during the follow-up in people who had recovered from pneumonia (log-rank test p<0.0001) (figure S3). Similarly, a significantly higher probability of cognitive impairment and dementia (as separate outcomes) was displayed in those with previous hospitalisation for pneumonia than in controls (log-rank test p<0.0001) (figure S3). An association between previous pneumonia and cognitive impairment and dementia was observed (adjusted hazard ratio (aHR) 1.53, 95% CI 1.46–1.61) (table 2). Identical effect sizes were found from the complete case and multiple imputation analysis (table S4). The higher risk for developing cognitive impairment and dementia was within 1 year after hospitalisation for pneumonia. During subsequent years, the risk decreased and remained almost stable. When cognitive impairment and dementia were examined separately, an increased risk was found for both conditions in people hospitalised for pneumonia; the effect was greater for dementia (aHR 2.04, 95% CI 1.74–1.95) than cognitive impairment (aHR 1.28, 95% CI 1.22 to 1.38).

**TABLE 2:** Incidence rates and hazard ratios (HR) for associations of cognitive impairment and/or dementia comparing people previously hospitalised for pneumonia and controls

	**Events**	**Rate per 1000 person-years**	**Unadjusted^#^** **HR (95% CI)**	**Adjusted^¶^** **HR (95% CI)**	**p-value**
**Overall, 0–16 years after hospitalisation**					
Either cognitive impairment or dementia					
** **Controls	10 650	13.2 (13.0–13.5)	1.00	1.00	
** **People hospitalised for pneumonia	2727	18.0 (17.3–18.7)	1.65 (1.57–1.73)	1.53 (1.46–1.61)	<0.0001
Cognitive impairment					
** **Controls	7014	8.7 (8.5–8.9)	1.00	1.00	
** **People hospitalised for pneumonia	1547	10.2 (9.7–10.7)	1.38 (1.30–1.47)	1.28 (1.22–1.38)	<0.0001
Dementia					
** **Controls	3636	4.5 (4.3–4.7)	1.00	1.00	
** **People hospitalised for pneumonia	1180	7.8 (7.4–8.3)	2.22 (2.05–2.41)	1.84 (1.74–1.95)	<0.0001
**Within 1** **year after hospitalisation**					
Either cognitive impairment or dementia					
** **Controls	2396	11.2 (10.8–11.7)	1.00	1.00	
** **People hospitalised for pneumonia	1071	22.9 (21.6–24.4)	2.04 (1.89–2.20)	1.89 (1.75–2.05)	<0.0001
Cognitive impairment					
** **Controls	1561	7.6 (7.3–8)	1.00	1.00	
** **People hospitalised for pneumonia	507	11.2 (10.3–12.3)	1.49 (1.34–1.65)	1.39 (1.25–1.55)	<0.0001
Dementia					
** **Controls	835	4.3 (4–4.6)	1.00	1.00	
** **People hospitalised for pneumonia	564	12.9 (11.9–14)	3.12 (2.77–3.51)	2.88 (2.56–3.26)	<0.0001
**>1**–**4** **years after hospitalisation**					
Either cognitive impairment or dementia					
** **Controls	4665	8.4 (8.2–8.6)	1.00	1.00	
** **People hospitalised for pneumonia	1073	9.7 (9.1–10.3)	1.43 (1.33–1.54)	1.33 (1.24–1.4)	<0.0001
Cognitive impairment					
** **Controls	3075	5.6 (5.4–5.8)	1.00	1.00	
** **People hospitalised for pneumonia	646	5.8 (5.4–6.3)	1.26 (1.15–1.39)	1.19 (1.08–1.32)	<0.001
Dementia					
** **Controls	1590	2.9 (2.8–3)	1.00	1.00	
** **People hospitalised for pneumonia	427	3.9 (3.5–4.3)	1.79 (1.58–2.03)	1.62 (1.43–1.84)	<0.0001
**>4**–**16** **years after hospitalisation**					
Either cognitive impairment or dementia					
** **Controls	3589	4.5 (4.3–4.6)	1.00	1.00	
** **People hospitalised for pneumonia	589	3.9 (3.6–4.2)	1.42 (1.31–1.63)	1.35 (1.20–1.50)	<0.0001
Cognitive impairment					
** **Controls	2378	2.9 (2.8–3.1)	1.00	1.00	
** **People hospitalised for pneumonia	394	2.6 (2.4–2.9)	1.47 (1.29–1.68)	1.35 (1.18–1.55)	<0.0001
Dementia					
** **Controls	1211	1.5 (1.4–1.6)	1.00	1.00	
** **People hospitalised for pneumonia	189	1.2 (1.1–1.4)	1.45 (1.18–1.79)	1.31 (1.06–1.63)	0.013

^#^: Cox model accounting for matched set (age, sex and practice); ^¶^: Cox model accounting for matched set (age, sex and practice) and adjusting for smoking, body mass index, alcohol consumption, depression, cerebrovascular diseases, type II diabetes, traumatic brain injury and hypertension.

### Stratified risk of cognitive impairment and dementia

Age modified the effect of previous pneumonia on cognitive impairment and dementia. Although younger people were at the lowest absolute risk of incident cognitive impairment and dementia, the effect of previous pneumonia was larger in younger people (p_interaction_<0.0001) with the size of the effect being similar for both age groups (18–44 and 45–60 years old) but statistical significance was only retained in the 45–60 year group (aHR 2.19; 95% CI 1.65–2.90) (table 3). Results were similar when the incidence of both conditions was examined separately. The aHRs stratified by sex and age groups for each outcome are reported in table S5.

**TABLE 3:** Incidence rates and hazard ratios (HR) for associations of cognitive impairment and/or dementia comparing people hospitalised for pneumonia and controls

**Variables**	**People hospitalised for pneumonia**		**Controls**		**Unadjusted^#^** **HR (95% CI)**	**Adjusted^¶^** **HR (95% CI)**	**p-value**
	**Events**	**Rate per 1000 person-years**	**Events**	**Rate per 1000 person-years**			
**Either cognitive impairment or dementia**							
Gender							0.256^§^
Male	1209	16.2 (15.3–17.1)	4612	11.4 (11.1–11.8)	1.68 (1.57–1.81)	1.51 (1.40–1.66)	<0.0001
Female	1518	19.8 (18.8–20.8)	6038	15 (14.6–15.4)	1.62 (1.52–1.73)	1.48 (1.36–1.59)	<0.0001
Age^+^, years							<0.0001^§^
18–44	40	1.4 (1–1.9)	74	0.6 (0.5–0.7)	2.25 (1.50–3.74)	1.84 (0.96–3.52)	0.0.063
45–60	142	4.6 (3.9–5.4)	278	1.9 (1.7–2.1)	2.66 (2.13–3.33)	2.19 (1.65–2.90)	<0.0001
61–74	560	13.6 (12.5–14.8)	1905	8.3 (7.9–8.6)	1.85 (1.66–1.98)	1.69 (1.50–1.92)	<0.0001
75–83	976	33 (31–35.2)	4167	22.8 (22.1–23.5)	1.65 (1.52–1.79)	1.46 (1.33–1.61)	<0.0001
>83	1009	47.2 (44.4–50.2)	4226	32.9 (31.9–33.9)	1.43 (1.31–1.55)	1.48 (1.27–1.41)	<0.0001
**Cognitive impairment**							
Gender							0.588^§^
Male	695	9.3 (8.6–10)	3216	8 (7.7–8.3)	1.38 (1.26–1.51)	1.29 (1.17–1.45)	<0.0001
Female	852	11.1 (10.4–11.9)	3798	9.5 (10.4–11.9)	1.39 (1.28–1.51)	1.28 (1.20–1.38)	<0.0001
Age^+^, years							<0.0001^§^
18–44	38	1.4 (1–1.8)	74	0.6 (0.5–0.8)	2.14 (1.42–3.23)	1.73 (0.90–3.33)	0.103
45–60	124	4 (3.4–4.8)	256	1.8 (1.6–2)	2.49 (1.97–3.15)	2.01 (1.57–2.82)	<0.0001
61–74	402	9.8 (8.9–10.8)	1531	6.6 (6.3–7)	1.62 (1.43–1.83)	1.55 (1.35–1.78)	<0.0001
75–83	568	19.2 (17.7–20.8)	2834	15.5 (14.9–16.1)	1.33 (1.20–1.48)	1.19 (1.06–1.35)	0.004
>83	415	19.4 (17.6–21.4)	2319	18.1 (17.3–18.8)	1.04 (0.92–1.18)	0.98 (0.85–1.14)	0.842
**Dementia**							
Gender							0.001^§^
Male	514	6.9 (6.3–7.5)	1396	3.5 (3.3–3.7)	2.46 (2.17–2.78)	2.12 (1.81–2.48)	<0.0001
Female	666	8.7 (8.1–9.4)	2240	5.6 (5.4–5.8)	2.08 (1.88–2.31)	1.91 (1.67–2.18)	<0.0001
Age^+^, years							<0.0001^§^
18–44	2	0.1 (0.02–0.2)	0	0	NA	NA	NA
45–60	18	0.6 (0.4–0.9)	22	0.2 (0.1–0.3)	5.21 (2.44–11.1)	2.10 (1.56–2.82)	<0.0001
61–74	158	3.8 (3.3–4.5)	374	1.6 (1.5–1.8)	2.94 (2.35–3.68)	2.39 (1.80–3.18)	<0.0001
75–83	408	13.8 (12.5–15.2)	1333	7.3 (6.9–7.6)	2.44 (2.12–2.80)	2.18 (1.85–2.60)	<0.0001
>83	594	27.8 (25.6–30.1)	1907	14.5 (14.2–15.5)	1.90 (1.70–2.13)	1.67 (1.43–1.95)	<0.0001

NA: not applicable. ^#^: Cox model accounting for matched set (age, sex and practice); ^¶^: Cox model accounting for matched set (age, sex and practice) and adjusting for smoking, body mass index, alcohol consumption, depression, traumatic brain injury, cerebrovascular diseases, type II diabetes and hypertension; ^+^: age at the index date; ^§^: p-value for interaction.

## Discussion

Our study highlights that people are significantly at increased risk of developing cognitive impairment and dementia after hospitalisation for pneumonia across all age groups. The highest risk of incident cognitive impairment and dementia was observed within a year of hospitalisation. Age modified the effect of pneumonia on cognitive impairment and dementia; although the absolute risk was highest in the older age groups, the size of the effect of increased risk was strongest in those aged 45–60 years.

Our results are in accordance with the limited current literature. Three previous studies that included older patients examined whether patients hospitalised for pneumonia have an increased risk of a decline in cognition or developing dementia. Davydow *et al.* [12] found increased odds (adjusted OR 2.46, 95% CI 1.60–3.79) of moderate-to-severe cognitive impairment in people aged ≥50 years or older who were hospitalised with pneumonia. Similarly, a study that included people aged ≥65 years found that participants with pneumonia were at increased risk of developing dementia (aHR 1.57, 95% CI 1.11–2.22) [13]. Tate *et al.* [14] reported that pneumonia hospitalisation was associated with a 1.9-fold (95% CI 1.40–2.80) increase in the hazard of dementia in older people. Finally, a small study (n=58) found that 23% of patients developed moderate to severe cognitive impairment within a year of hospitalisation for community-acquired pneumonia [24]. Our study extends the literature by providing estimates for the risk of cognitive impairment and dementia, which are lacking, while stratifying by follow-up interval, sex and age groups.

The mechanisms by which pneumonia may increase the risk of cognitive impairment and dementia are multifactorial. Systemic infection including pneumonia is a well-recognised risk factor for delirium [25]. Activation of microglia is key for the mediation of the behavioural effects of systemic infections. Although the microglial response is usually tightly regulated to prevent deleterious effects, the self-propelling defensive features could become neurotoxic, reducing the threshold for delirium. Despite initial recovery from delirium, findings from follow-up studies confirm an association between delirium and long-term cognitive impairment and dementia, suggesting longer-lasting cognitive impacts may occur [26, 27]. Studies are emerging regarding the longer-term effects of severe COVID-19 on cognitive function. Cognitive impairment can still be observed 1 year after hospital discharge due to COVID-19 [28]. Whether the underlying mechanisms behind longer-term cognitive impairment from pneumonia and from COVID-19 are similar awaits further investigation.

Alternatively, it is possible that hospitalisation with pneumonia is a marker for subclinical or undiagnosed cognitive impairment or dementia [29]. Pneumonia-related hypoxia [30] and inflammation [31, 32] may contribute to subsequent cognitive impairment and dementia. These different reasons for the observed association may all contribute in part and may be differentially important across different ages.

We found stronger associations between pneumonia, cognitive decline and dementia in the short term than in the long term. Reverse causation and ascertainment bias can contribute to short-term associations, but infection might also accelerate or exacerbate existing neuropathology [13, 33, 34]. Robust, but weaker, long-term associations suggest that pneumonia infection might also trigger early stages of neurodegeneration.

Cognitive impairment and dementia in older people are well recognised and confirmed in our data. That the risk was also observed in people aged 45–60 years hospitalised for pneumonia is also important as they are not typically regarded as being at high risk of cognitive impairment or dementia. Awareness of this propensity in this age group following pneumonia may enable more timely appropriate clinical management, such as preventative measures, or earlier diagnosis.

This is the first large study to observe that the risk of cognitive impairment following pneumonia is greater for younger persons than for older persons. Possible reasons for this observation include: 1) younger persons who develop pneumonia severe enough to require hospital care have host characteristics that also place them at higher risk of developing cognitive impairment, which has not been measured in the current dataset (residual confounding); 2) the degree of systemic inflammation may have been higher in these younger patients than in older patients and the degree of systemic inflammation may be associated with future cognitive impairment; 3) the pathogens causing pneumonia in younger persons may have been different and different pathogens may be associated differentially with subsequent cognitive impairment. These explanations are speculative until further investigated.

A key strength of this study is the large sample size that is representative of the population in England, UK. We captured the cognitive impairment and dementia diagnoses for the general pneumonia population, not limited to a specific subset, such as older people. There are a few study limitations that warrant discussion. First, although considerable efforts were taken to ensure data quality, we cannot fully exclude the possibility of any diagnosis misclassification as we were reliant on how accurately general practitioners recorded these conditions. Historically, dementia has been underdiagnosed by ≥50% but that has improved since the National Dementia Strategy in 2009, and especially since 2015 when diagnosis and dementia registers have been incentivised. In many places, dementia ascertainment rates are >80% of that expected epidemiologically. In this study, no differential bias is expected as both patients with pneumonia and controls were identified using the same methodology. Second, a significant proportion of people hospitalised with pneumonia consult primary care after discharge. This may increase the opportunities for a diagnosis of cognitive impairment and dementia in those people compared to controls. Third, owing to the nature of the data, we were unable to assess the degree of severity of the diseases. Finally, the absence of education as a variable in the model is a recognised limitation, although it is unlikely that this single variable would dramatically alter the main study findings given the very large size of the sample, together with adjustment for all major confounders.

Our findings suggest post-discharge care plans by clinicians, in partnership with patients, aimed at preventative measures are warranted. These could include active screening and primary prevention strategies targeted at modifiable risk factors for cognitive impairment and dementia. Major risk factors for dementia are identified in early life (low level of education), midlife (hypertension, obesity, hearing loss, traumatic brain injury and alcohol misuse) and later life (smoking, depression, physical inactivity, social isolation, diabetes and air pollution) can contribute to decreased dementia risk [19]. Additionally, increased attention to the prevention of pneumonia, both as the first consideration and following a first episode, are also justified, particularly in those patients at higher risk of pneumonia. These measures include pneumococcal, seasonal influenza and COVID-19 vaccinations (when indicated).

In conclusion, adults who recover following hospitalisation for pneumonia have an increased risk of a new diagnosis of cognitive impairment or dementia, particularly in the subsequent 12 months, compared to the general population. Further research is needed to better understand the underlying pathophysiological mechanisms for this association and this information will be important in identifying appropriate future interventions. In the meantime, evidence-based preventative measures against dementia should be promoted in patients recovering from pneumonia, particularly for younger men who may not perceive themselves to be at higher risk.

## Supplementary material

10.1183/23120541.00328-2022.Supp1 **Please note:** supplementary material is not edited by the Editorial Office, and is uploaded as it has been supplied by the author.
Supplementary material 00328-2022.SUPPLEMENT
