# Supplementary material for: Incidence of cognitive impairment and dementia after hospitalisation for pneumonia: a UK population-based matched cohort study
Source: ERJ Open Res. 2023 May 9;9(3):00328-2022. doi: 10.1183/23120541.00328-2022 (PMC10204809; doi:10.1183/23120541.00328-2022)
Supplement: Supplementary file 1 [file 00328-2022.SUPPLEMENT.pdf]

Incidence of cognitive impairment and dementia after hospitalisation for pneumonia: a UK population-based matched cohort study

Christos V. Chalitsios, Vadsala Baskaran, Rowan H. Harwood, Wei Shen Lim, Tricia M. McKeever.

Table of Contents

DIAGNOSTIC CODES 2

METHODS 7

RESULTS 8

SUPPLEMENTS

Diagnostic codes

Table S1. ICD-10 codes for pneumonia.

ICD-10 code	Description
**J12**	Viral pneumonia, not elsewhere classified
**J13**	Pneumonia due to *Streptococcus pneumoniae*
**J14**	Pneumonia due to *Haemophilus influenzae*
**J15**	Bacterial pneumonia, not elsewhere classified
**J16**	Pneumonia due to other infectious organisms, not elsewhere classified
**J17**	Pneumonia in diseases classified elsewhere
**J18**	Pneumonia, unspecified organism

Table S2. Medcodes for cognitive impairment.

Medcode	Description
**1993**	memory loss - amnesia
**3639**	amnesia symptom
**5777**	memory loss symptom
**2908**	memory disturbance
**27788**	temporary loss of memory
**103453**	short-term memory loss
**39507**	poor visual sequential memory
**40821**	poor auditory sequential memory
**67163**	disturbance of memory for order of events
**110307**	memory impairment
**7674**	cognitive decline
**107282**	mild cognitive impairment
**107482**	moderate cognitive impairment
**107402**	severe cognitive impairment
**108266**	cognitive impairment
**52947**	memory: own age not known
**53146**	memory: present time not known
**53014**	memory: present place not known
**52948**	memory: present year not known
**53125**	memory: own dob not known
**52825**	memory: present month not known
**52800**	memory: important event not known
**52801**	memory: important person not known
**52805**	memory: count down unsuccessful
**53016**	memory: address recall unsuccessful
**65856**	gds level 2 - very mild cognitive decline
**60263**	gds level 3 - mild cognitive decline
**60726**	gds level 4 - moderate cognitive decline
**70057**	gds level 5 - moderately severe cognitive decline

**94717**	gds level 6 - severe cognitive decline
**72520**	gds level 7 - very severe cognitive decline
**6387**	mild memory disturbance
**6061**	organic memory impairment
**11936**	[x]mild cognitive disorder
**110729**	[x]cognitive communication disorder
**31572**	visual disorientation syndrome
**7711**	[d]memory deficit
**20683**	[d]disorientation, unspecified
**112378**	[x]symptoms/signs involving cognition, percept, emotion state & behaviour
**52939**	[x]other & unspecified symptom/signs involving cognitive function/awareness
**52811**	[x]disorientation, unspecified
**10822**	impaired cognition
**61639**	unable to recognise surroundings
**47279**	mistakes people's identity
**67565**	does not recognise self
**61869**	does not recognise photographs of self
**92635**	unable to recognise parts of own body
**101458**	unable to recognise objects
**100788**	unable to recognise faces
**91516**	unable to recognise familiar people
**59539**	unable to reason
**52550**	difficulty reasoning
**46554**	unable to use verbal reasoning
**46320**	difficulty using verbal reasoning
**99474**	difficulty using visuospatial reasoning
**48506**	unable to process information
**50446**	difficulty processing information
**61308**	unable to process information accurately
**57609**	difficulty processing information accurately
**109311**	unable to process information at normal speed
**99588**	difficulty processing information at normal speed
**56044**	unable to analyse information
**57608**	difficulty analysing information
**50843**	difficulty performing logical sequencing
**66172**	isolated memory skills
**19719**	orientation confused
**64219**	orientation poor
**66012**	disorientation for person
**55460**	spatial disorientation
**51379**	memory disturbance (& amnesia (& symptom))
**67838**	memory loss symptom
**103375**	memory loss - amnesia

**105538**	memory disturbance
**102880**	loss of memory
**10123**	memory loss
**68230**	memory gone
**12805**	memory loss - amnesia
**19297**	loss of memory
**12277**	lom - loss of memory
**32367**	impairment of working memory
**65696**	impairment of primary memory
**37191**	poor memory for remote events
**9786**	loss of memory for recent events
**67802**	no memory for recent events
**67998**	temporary loss of memory
**47882**	transient memory loss
**10514**	memory impairment
**39915**	memory dysfunction
**50418**	memory deficit
**26434**	bad memory
**12057**	memory problem
**12583**	poor memory
**19073**	memory lapses
**51739**	distortion of memory
**64892**	invents experiences to compensate for loss of memory
**11410**	poor short-term memory
**10571**	short-term memory loss
**53978**	poor long-term memory
**47581**	long-term memory loss
**98798**	delayed verbal memory
**46860**	difficulty making plans
**46564**	difficulty making decisions
**53388**	unable to use decision-making strategies
**107021**	difficulty using decision-making strategies
**65319**	unable to make considered choices
**43204**	difficulty making considered choices
**59242**	difficulty solving problems
**40002**	language-related cognitive disorder

Table S3. Medcodes for dementia diagnosis.

Medcode	Description
**1916**	Senile dementia
**1350**	Senile/presenile dementia
**7323**	Uncomplicated senile dementia
**15165**	Presenile dementia
**42602**	Uncomplicated presenile dementia
**30032**	Presenile dementia with paranoia
**27677**	Presenile dementia with depression
**38438**	Presenile dementia NOS
**44674**	Senile dementia with depressive or paranoid features
**18386**	Senile dementia with paranoia
**21887**	Senile dementia with depression
**41089**	Senile dementia with depressive or paranoid features NOS
**37015**	Senile dementia with delirium
**19477**	Arteriosclerotic dementia
**43089**	Uncomplicated arteriosclerotic dementia
**55467**	Arteriosclerotic dementia with paranoia
**43292**	Arteriosclerotic dementia with depression
**42279**	Arteriosclerotic dementia NOS
**25386**	Dementia in conditions EC
**4951**	Chronic confusional state
**7664**	[X]Dementia in Alzheimer's disease
**49263**	[X]Dementia in Alzheimer's disease with early onset
**25704**	[X]Presenile dementia,Alzheimer's type
**60059**	[X]Primary degen dementia, Alzheimer's type, presenile onset
**61528**	[X]Alzheimer's disease type 2
**38678**	[X]Dementia in Alzheimer's disease with late onset
**46762**	[X]Alzheimer's disease type 1
**11379**	[X]Senile dementia,Alzheimer's type
**43346**	[X]Primary degen dementia of Alzheimer's type, senile onset
**30706**	[X]Dementia in Alzheimer's dis, atypical or mixed type
**29386**	[X]Dementia in Alzheimer's disease, unspecified
**8195**	[X]Alzheimer's dementia unspec
**6578**	[X]Vascular dementia
**9565**	[X]Arteriosclerotic dementia
**46488**	[X]Vascular dementia of acute onset
**55838**	[X]Predominantly cortical dementia
**8934**	[X]Subcortical vascular dementia
**31016**	[X]Mixed cortical and subcortical vascular dementia
**55313**	[X]Other vascular dementia
**19393**	[X]Vascular dementia, unspecified
**12621**	[X]Dementia in other diseases classified elsewhere

**28402**	[X]Dementia in Pick's disease
**26270**	[X]LEWY BODY DEMENTIA
**64267**	[X]Dementia in other specified diseases classif elsewhere
**4693**	[X] Unspecified dementia
**48501**	[X] Presenile dementia NOS
**34944**	[X] Primary degenerative dementia NOS
**4357**	[X] Senile dementia NOS
**27759**	[X] Senile dementia, depressed or paranoid type
**1917**	ALZHEIMER'S DISEASE
**16797**	ALZHEIMER'S DISEASE WITH EARLY ONSET
**32057**	ALZHEIMER'S DISEASE WITH LATE ONSET
**11136**	pick's disease
**29512**	senile degeneration of brain
**7572**	lewy body disease
**59122**	[x]other alzheimer's disease
**8634**	Multi infarct dementia
**11175**	[X]Multi-infarct dementia
**9509**	[X]Dementia in Parkinson's disease
**68125**	[x]delirium not superimposed on dementia, so described
**112783**	[x]delirium of mixed origin
**25066**	[x]delirium, not induced by alcohol+other psychoactive subs
**53924**	[x]delirium, unspecified
**52394**	[x]other delirium
**22466**	delirium - acute organic
**24077**	delirium - subacute organic
**5367**	o/e - delirious
**49513**	presenile dementia with delirium
**53446**	[x]delirium superimposed on dementia
**56912**	arteriosclerotic dementia with delirium

Methods

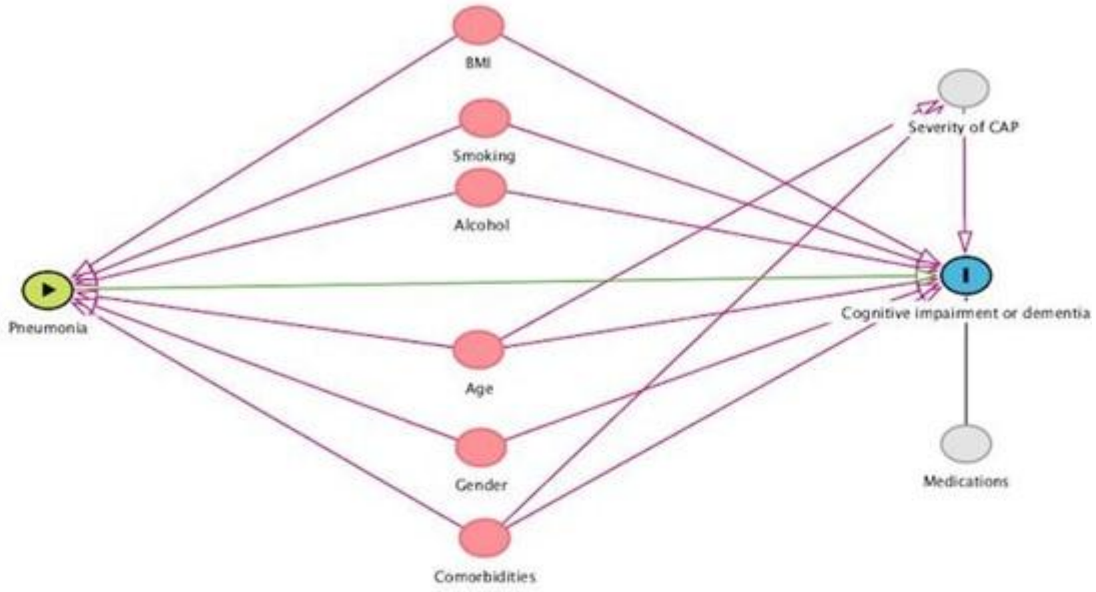

Figure S1. Directed acyclic graph illustrating the association between admission for pneumonia (exposure) and developing cognitive impairment and dementia (outcome).

Defining smoking status

Smoking status was divided into three categories: never smokers, ex-smokers, and current smokers. If patients had more than one record for smoking status, the most recent record of smoking status was used. Never smokers were reclassified to ex-smokers if they had any record of smoking recorded in their entire clinical record entered on CPRD prior to study entry. Read code lists for smoking status were developed using validated medical Read codes.

Defining alcohol status

Self-reported alcohol consumption was collected prospectively and coded by general practitioners or practice nurses on the consultation date in CPRD. The most recent alcohol consumption record prior the index date was used to classify participants drinking behaviour. Four categories were defined including: (1) non-drinkers (Read codes such as "Non-drinker alcohol"), (2) former drinkers (Read codes such as "stopped drinking alcohol"), (3) occasional drinkers (Read codes such as "drinks rarely"), and (4) current drinkers (Read codes such as “drinks wine”, and “alcohol misuse”). Data based on the alcohol status and the alcohol units per week from the additional file of CPRD were also extracted to define patients in the above categories, where available. The information about the alcohol status helped to include more patients as “non-drinkers” or “former-drinkers”, and if a patient had more than 0 alcohol units per week classified as “current drinker”. Non-drinkers were reclassified as former drinkers if they had any record of drinking

recorded in their entire clinical record entered on CPRD prior to study entry, otherwise their category remained the same.

Power study calculation

Outcome: Dementia/Cognitive impairment	Ratio of unexposed to exposed	Hazard ratio (unexposed v. Exposed)	Exposed patients	Unexposed patients
**Exposure: Hospitalisation for pneumonia**	4	1.10	2,617	10,468

Standard assumptions:

Powered at 90% with a type 1 error probability of 0.05

Median survival time in unexposed cases is assumed to be 6 years (based on previous work in this topic area) with the ratio of unexposed to exposed subjects 4:1; accrual time of 5 years and follow-up of 1 year

Hazard ratios based on previous point estimates obtained from scientific literature and consultation with study collaborators considering the clinical significance

Results

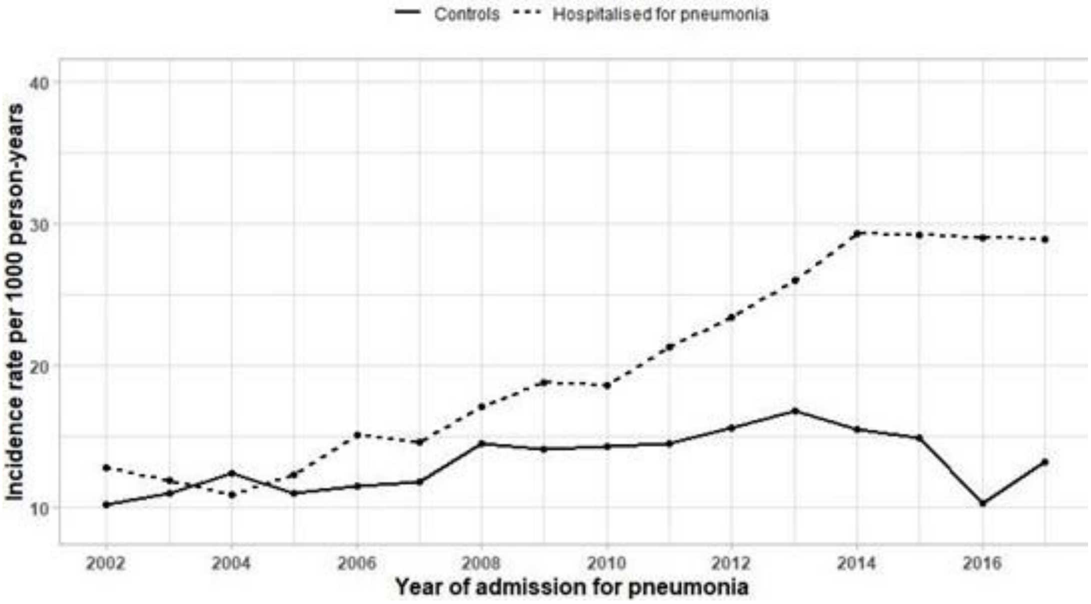

Figure S2. Incidence rate for cognitive impairment and dementia events by year of admission for pneumonia.

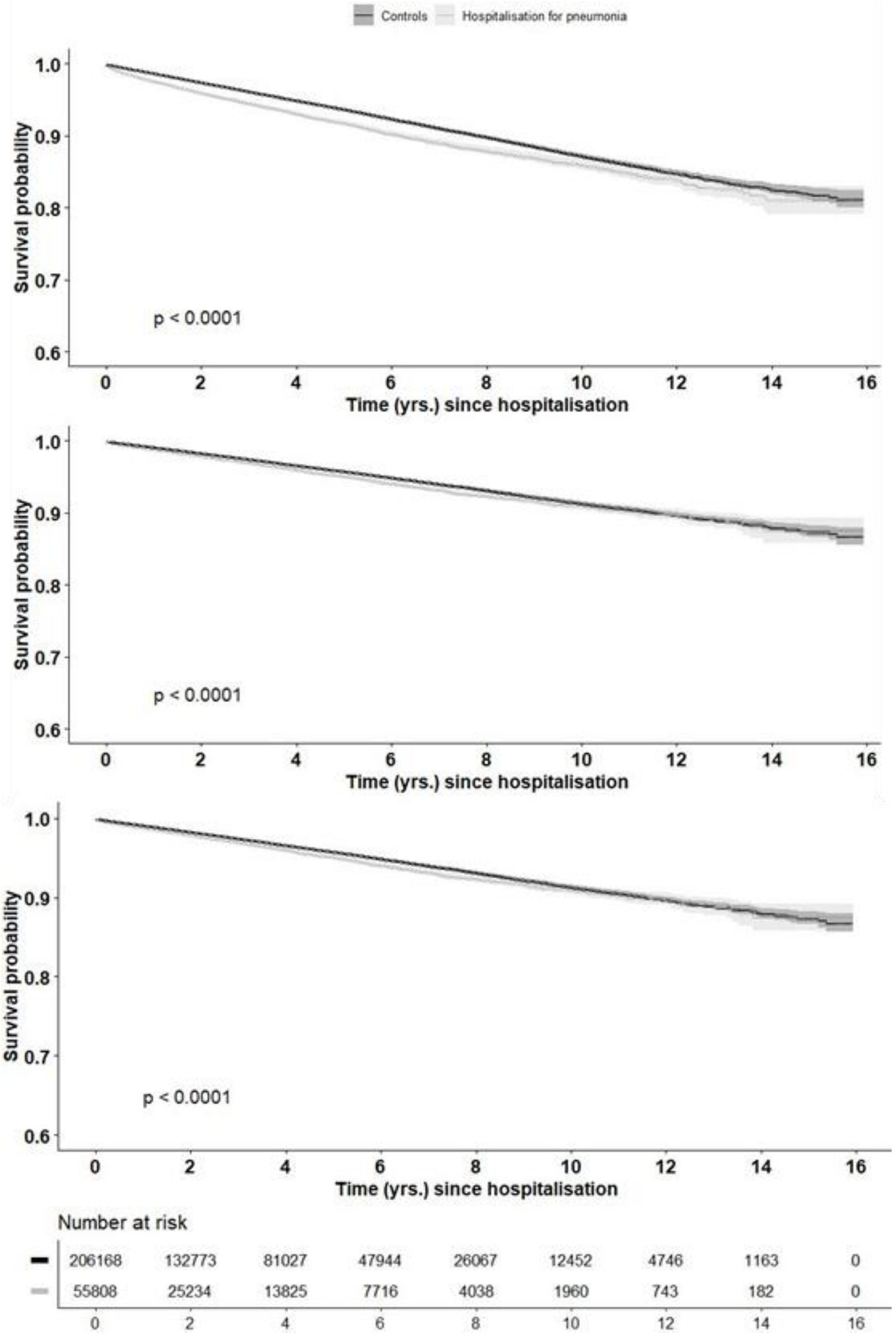

Figure S3. Kaplan-Meier plot with 95%CI and the log-rank test of incidence of time post hospitalisation to first either cognitive impairment or dementia episode (top), first cognitive impairment episode (middle), first dementia episode (bottom) in people previously hospitalised for pneumonia and controls.

Table S4. Adjusted hazard ratios (aHR) for the association of cognitive impairment and/or dementia comparing people previously hospitalised for pneumonia and controls after a complete case and multiple imputation analysis.

	**Either cognitive impairment or dementia**		**Cognitive impairment**	
**Variables**	**Adjusted HR[1] (95%CI)**	**P value**	**Adjusted HR[1] (95%CI)**	**P value**
**Either cognitive impairment or dementia Males**				
Controls	1.00		1.00	
People hospitalised for pneumonia	1.49 (1.41-1.58)	<.0001	1.55 (1.48-1.63)	<.0001
**Cognitive impairment**				
Controls	1.00		1.00	
People hospitalised for pneumonia	1.29 (1.20-1.38)	<.0001	1.32 (1.24-1.40)	<.0001
**Dementia**				
Controls	1.00		1.00	
People hospitalised for pneumonia	2.01 (1.81-2.22)	<.0001	2.07 (1.91-2.25)	<.0001

[2] *Cox model accounting for matched set (age, sex, and practice) and adjusting for smoking, body mass index, alcohol consumption, depression, cerebrovascular diseases, type II diabetes, traumatic brain injury, and hypertension*

Table S5. Hazard ratios (HR) for association of cognitive impairment and dementia with exposure to hospitalisation for pneumonia stratified by gender and age groups.

	**Either cognitive impairment or dementia**		**Cognitive impairment**		**Dementia**	
**Variables**	**Adjusted HR (95%CI)**	**P value**	**Adjusted HR (95%CI)**	**P value**	**Adjusted HR (95%CI)**	**P value**
			**Males**			
**Age[1]**						
**18-44**	0.98 (0.25-3.88)	.98	0.89 (0.22-3.55)	.867	NA	
**45-60**	2.42 (1.61-3.63)		2.18 (1.41-3.38)	<.001	2.03 (0.51-8.32)	.323
**61-74**	1.65 (1.41–1.92)	<.0001	1.46 (1.22-1.75)	<.0001	2.60 (1.86-3.66)	<.0001
**75-83**	1.52 (1.33-1.72)	<.0001	1.27 (1.09-1.50)	.002	2.15 (1.73-2.68)	<.0001
**>83**	1.35 (1.17-1.55)	<.0001	0.89 (0.72-1.09)	.254	1.98 (1.64-2.41)	<.0001
**Age[1]**						
**18-44**	2.84 (1.24-6.47)	.014	2.65 (1,15-6.10)	.022	NA	
**45-60**	1.97 (1.32-2.94)	<.001	1.97 (1.30-2.98)	.001	1.29 (0.90-1.86)	.851
**61-74**	1.74 (1.47-2.05)	<.0001	1.53 (1.27-1.85)	<.0001	2.60 (1.82-3.70)	<.0001
**75-83**	1.54 (1.33-1.70)	<.0001	1.26 (1.10-1.47)	.002	2.11 (1.75-2.57)	<.0001
**>83**	1.38 (1.25-1.54)	<.0001	1.08 (0.92-1.26)	.347	1.74 (1.50-2.01)	<.0001

[1] *Age at the index date.*

[2] *Cox model accounting for matched set (age, sex, and practice) and adjusting for smoking, body mass index, alcohol consumption, depression, cerebrovascular diseases, type II diabetes, traumatic brain injury, and hypertension*
